# P-2036. Obeldesivir for Treatment of COVID-19 in Adults and Adolescents Without Risk Factors for Progression to Severe Disease: the OAKTREE Study

**DOI:** 10.1093/ofid/ofae631.2192

**Published:** 2025-01-29

**Authors:** Onyema Ogbuagu, Jason D Goldman, Robert L Gottlieb, Upinder Singh, Masaharu Shinkai, Gerard Acloque, Dahlene Fusco, Erika Gonzalez, Princy N Kumar, Annie Luetkemeyer, Amos Lichtman, Afsaneh Mozaffarian, Yiannis Koullias, Robert H Hyland, Joe Llewellyn, Anu Osinusi, Frank Duff, Rita Humeniuk, Luzelena Caro, Santosh Davies, Charlotte Hedskog, Shuguang Chen, Kim Etchevers, Priyanka Nadig, Anita Kohli

**Affiliations:** Yale School of Medicine, Cheshire, CT; Swedish Medical Center, Seattle, WA, USA, and Division of Allergy and Infectious Diseases, University of Washington, Seattle, WA; Baylor University Medical Center, Dallas, Texas; Stanford University, CA; Tokyo Shinagawa Hospital, Tokyo, Tokyo, Japan; Universal Medical and Research Center, LLC, Coral Gables, Florida; Tulane School of Medicine, New Orleans, Louisiana; STAAMP Research, San Antonio, Texas; Georgetown University Medical Center, Washington, DC, USA, Washington, District of Columbia; University of California San Francisco, San Francisco, CA; Gilead Sciences, Inc., Foster City, California; Gilead Sciences, Inc., Foster City, California; Gilead Sciences, Inc., Foster City, California; Gilead Sciences, Inc., Foster City, California; Gilead Sciences, Inc., Foster City, California; Gilead, Foster City, California; Gilead Sciences, Inc, San Francisco, California; Gilead Sciences, Inc., Foster City, California; Gilead Sciences, Inc., Foster City, California; Gilead Sciences, Inc., Foster City, California; Gilead Sciences, Inc., Foster City, California; Gilead Sciences, Inc, San Francisco, California; Gilead Sciences, Inc., Foster City, California; Gilead Sciences, Inc., Foster City, California; Arizona Liver Health, Tucson, Arizona

## Abstract

**Background:**

COVID-19 continues to cause morbidity. Obeldesivir (ODV) is an oral, broad spectrum, nucleoside analog prodrug inhibitor of SARS-CoV-2 RNA-dependent RNA polymerase.

**Methods:**

OAKTREE was a Phase 3, randomized, double-blind, placebo (PBO)-controlled study in adults and adolescents without risk factors for progression to severe COVID-19. Participants were enrolled within 3 days of COVID-19 symptom onset and randomized 1:1 to receive 350 mg of ODV or PBO twice a day for 5 days. Primary endpoints were time to symptom alleviation (9 symptoms; first of 48 consecutive hours) by Day 29 and incidence of adverse events (AEs) and laboratory abnormalities. Secondary endpoints included symptom resolution and viral kinetics.

**Results:**

1955 participants were enrolled (Feb 13-Oct 31, 2023), randomized, and received ≥1 dose of study drug (98% completed study drug and the study). Baseline characteristics were similar between groups (overall mean age 41 years, 59% female, 93% Hispanic or Latino, 87% White, 11% Black, and 2% Asian). Seropositivity was >99% for SARS-CoV-2 (anti-S or anti-N) antibodies, 73% were vaccinated, and mean baseline SARS-CoV-2 viral load was 5.09 log_10_ copies/mL. The efficacy analysis set consisted of 1768 participants with PCR-confirmed SARS-CoV-2 at baseline. The Kaplan-Meier estimate of median time to symptom alleviation was 5.9 days (95% CI, 5.4-6.1) with ODV and 6.0 days (95% CI, 5.8-6.3) with PBO (*P* = 0.068). Times to symptom resolution were similar (ODV: 9.2 days [95% CI 8.9-10.0]; PBO: 9.3 days [95% CI 8.9-10.1]; *P* = 0.56). ODV reduced viral load at Days 3 and 5, with least squares mean treatment differences of -0.31 (*P* < 0.0001) and -0.18 (*P* = 0.0037) log_10_ copies/mL, respectively. The safety profile of ODV was similar to PBO, with similar rates of AEs, treatment-related AEs, serious AEs, and AEs leading to study drug discontinuation. No deaths or hospitalizations were reported by Day 29.

**Conclusion:**

ODV was generally safe and well tolerated but did not significantly reduce time to symptom alleviation or resolution in a standard risk population with >99% SARS-CoV-2 seropositivity. ODV reduced SARS-CoV-2 viral load at Days 3 and 5 compared to PBO. Symptom duration was markedly shorter than in prior trials, consistent with milder disease in a population with high rates of hybrid immunity.

**Disclosures:**

Onyema Ogbuagu, MD, Gilead Sciences, Inc.: Advisor/Consultant|Gilead Sciences, Inc.: Honoraria|GSK/ViiV: Advisor/Consultant|GSK/ViiV: Honoraria|Janssen: Advisor/Consultant|Moderna: Advisor/Consultant|Moderna: Honoraria Jason D. Goldman, MD, MPH, Gilead Sciences, Inc.: Advisor/Consultant|Gilead Sciences, Inc.: Grant/Research Support|Gilead Sciences, Inc.: Speaker|Helix: Grant/Research Support Robert L. Gottlieb, MD, AbbVie: Advisor/Consultant|AbCellera: Stocks/Bonds (Public Company)|AstraZeneca: Advisor/Consultant|Eli Lilly: Advisor/Consultant|Gilead Sciences Inc.: Advisor/Consultant|Gilead Sciences Inc.: Honoraria|Gilead Sciences Inc.: travel support, gift-in-kind to his institution to facilitate an unrelated academic-sponsored clinical trial NCT03383419|GSK Pharmaceuticals: Advisor/Consultant|Johnson & Johnson: Advisor/Consultant|Pfizer: Honoraria|Regeneron: Grants or contracts to institution|Roche: Advisor/Consultant|Roivant Sciences (Kinevant Sciences): Grants or contracts to institution Upinder Singh, MD, FIDSA, Gilead Sciences, Inc.: Advisor/Consultant|Pfizer: Grant/Research Support|Regeneron: Advisor/Consultant Masaharu Shinkai, MD, PhD, AstraZeneca: Grant/Research Support|AstraZeneca: Lecture fees|Fujifilm: Grant/Research Support|Genova Inc: Grant/Research Support|Gilead Sciences, Inc.: Grant/Research Support|GSK: Grant/Research Support|GSK: Lecture fees|Kyorin Pharmaceutical: Lecture fees|Pfizer: Grant/Research Support|Sanofi: Grant/Research Support|Sanofi: Lecture fees|Shionogi: Grant/Research Support Dahlene Fusco, MD, PhD, AICURIS: Site PI|Gilead Sciences, Inc.: Advisor/Consultant|Gilead Sciences, Inc.: Site PI|Metro Biotech: Site PI|Regeneron: Site PI|The Biomedical Advanced Research and Development Authority (BARDA) and the National Institute of Allergy and Infectious Diseases (NIAID): Site PI Erika Gonzalez, MD, AstraZeneca: Clinical research PI; Business relationship|DBV: Clinical research PI; Business relationship|Genentech: Clinical research PI; Business relationship|Novartis: Clinical research PI; Business relationship|Regeneron: Clinical research PI; Business relationship Princy N. Kumar, MD, Gilead Sciences, Inc.: Advisor/Consultant|Gilead Sciences, Inc.: Board Member|Gilead Sciences, Inc.: Grant/Research Support|Gilead Sciences, Inc.: Medical writing support provided by Aspire Scientific (Bollington, UK)|Gilead Sciences, Inc.: Stocks/Bonds (Public Company)|Johnson & Johnson: Stocks/Bonds (Public Company)|Merck: Advisor/Consultant|Merck: Board Member|Merck: Grant/Research Support|Merck: Stocks/Bonds (Public Company)|Moderna: Stocks/Bonds (Public Company)|Pfizer: Stocks/Bonds (Public Company)|Theratechnologies: Grant/Research Support|ViiV/GSK: Advisor/Consultant|ViiV/GSK: Board Member|ViiV/GSK: Grant/Research Support|ViiV/GSK: Stocks/Bonds (Public Company) Annie Luetkemeyer, MD, Cepheid: Grant/Research Support|Gilead Sciences, Inc.: Grant/Research Support|GSK: Grant/Research Support|Merck: Grant/Research Support|ViiV: Grant/Research Support|Vir: Advisor/Consultant Amos Lichtman, MPH, MD, Gilead Sciences, Inc.: Employee|Gilead Sciences, Inc.: Stocks/Bonds (Public Company) Afsaneh Mozaffarian, MS, Gilead Sciences, Inc.: Employee|Gilead Sciences, Inc.: Stocks/Bonds (Public Company) Yiannis Koullias, MD, Gilead Sciences, Inc.: Employee|Gilead Sciences, Inc.: Stocks/Bonds (Public Company) Robert H. Hyland, DPhil, Gilead Sciences, Inc.: Employee|Gilead Sciences, Inc.: Stocks/Bonds (Public Company) Joe Llewellyn, PharmD, Gilead Sciences, Inc.: Employee|Gilead Sciences, Inc.: Stocks/Bonds (Public Company) Anu Osinusi, MD, Gilead Sciences, Inc.: Employee|Gilead Sciences, Inc.: Stocks/Bonds (Public Company) Frank Duff, MD, Gilead Sciences, Inc.: Employee|Gilead Sciences, Inc.: Stocks/Bonds (Public Company) Rita Humeniuk, PhD, Gilead Sciences, Inc.: Former Employee|Gilead Sciences, Inc.: Stocks/Bonds (Public Company) Luzelena Caro, PhD, Gilead Sciences, Inc.: Employee|Gilead Sciences, Inc.: Stocks/Bonds (Public Company) Santosh Davies, MD, Gilead Sciences, Inc.: Employee|Gilead Sciences, Inc.: Stocks/Bonds (Public Company) Charlotte Hedskog, PhD, Gilead Sciences, Inc.: Employee|Gilead Sciences, Inc.: Stocks/Bonds (Public Company) Shuguang Chen, PhD, Gilead Sciences, Inc.: Employee|Gilead Sciences, Inc.: Stocks/Bonds (Public Company) Kim Etchevers, MS, Gilead Sciences, Inc.: Employee|Gilead Sciences, Inc.: Stocks/Bonds (Public Company) Priyanka Nadig, MPH, Gilead Sciences, Inc.: Employee|Gilead Sciences, Inc.: Stocks/Bonds (Public Company) Anita Kohli, MD, Eli Lilly: Received clinical trial revenue|Gilead Sciences, Inc.: Advisor/Consultant|Gilead Sciences, Inc.: Received clinical trial revenue|Vir: Received clinical trial revenue

